# Lay Health Workers in Community-Based Care and Management of Dementia: A Qualitative ‘Pre' and ‘Post' Intervention Study in Southwestern Uganda

**DOI:** 10.1155/2022/9443229

**Published:** 2022-03-23

**Authors:** Christine K. Karungi, Edith K. Wakida, Godfrey Z. Rukundo, Zohray M. Talib, Jessica E. Haberer, Celestino Obua

**Affiliations:** ^1^Office of Research Administration, Mbarara University of Science and Technology, P. O. Box 1410, Mbarara, Uganda; ^2^Department of Psychiatry, Mbarara University of Science and Technology, Mbarara, Uganda; ^3^Department of Medical Education, California University of Science and Medicine, San Bernardino, USA; ^4^Massachusetts General Hospital Center for Global Health and Harvard Medical School, Boston, USA; ^5^Office of the Vice Chancellor, Mbarara University of Science and Technology, Mbarara, Uganda

## Abstract

**Background:**

The global need for efficient and cost-effective use of healthcare resources in low-income countries has led to the introduction of lay health workers (LHWs) as a link of the community to healthcare services. As such, the LHWs perform a variety of tasks such as education, support for care delivery, and social support across all disease types. However, little is known about their ability to support dementia care and management in the community.

**Purpose:**

The goal of the pilot intervention was to evaluate the 5-day training intervention for LHWs in rural southwestern Uganda in community-based care and management of people with dementia, and implementation of the knowledge and skills gained.

**Methods:**

This was a “pre” and “post” pilot intervention study which involved a qualitative assessment of LHWs' knowledge on community-based management and care for people with dementia. We focused on four core competency domains in the WHO dementia toolkit. The intervention included a five-day training of the LHWs on dementia care, eight weeks of implementation, and an evaluation of the experiences. Analysis focused on the needs assessment, early detection and management, community engagement, support for people with dementia; and evaluation of the eight weeks implementation.

**Results:**

Before the training, the LHWs did not know much about what dementia-related support to provide in the community. Activities were limited to general support, including nutrition, and health education. After the training, LHWs had a basic understanding of dementia and began sensitizing the communities. They felt more comfortable working with people with dementia and reported a notable change in the attitude of family members. However, they reported challenges in differentiating the signs of early dementia from superstitious beliefs.

**Conclusion:**

With enhanced capacity, LHWs may be able to support community-based management for people with dementia. A larger study is needed to explore potential roles for LHWs and further assess effectiveness of the LHWs' skills.

## 1. Background

The global need for efficient and cost-effective use of healthcare resources in low-income countries has led to the introduction of lay health workers (LHWs) as a link of the community to the formal health services, especially in remote areas [1, 2]. The LHWs perform a variety of tasks and roles such as patient care, education, support for care delivery, care coordination, and social support across all disease types [1]. As such, there is an increased demand for a similar approach for mental health service provision where there is a vast shortage of mental health professionals relative to the burden of mental, neurological, and substance use disorders (MNS) [3] including dementia [4].

Given the rapidly increasing number of people living with dementia (5-8% of the general population aged 60 years and over) with no current cure, community-based support is needed to improve the lives of people with dementia, their caregivers, and families [5, 6]. Dementia is a global public health concern with an estimated 50 million people living with it worldwide [7], with a projected increase to 82 million in 2030 and 152 million in 2050 [5]. Much of the increase (75%) is attributable to the rising numbers of people with dementia living in low- and middle-income countries (LMICs) [5, 8], thus a call for action for the communities to be linked to the health systems in these settings.

In 2018, the World Health Organization (WHO) introduced the dementia toolkit for community health workers in LMICs [9]. The purpose of the tool was to help community workers (LHWs) gain skills and build capacity in identifying people at risk for dementia and make referrals when necessary; provide support to people with dementia, their families, and caregivers; and engage local communities in dementia-related activities and contribute to the development of dementia-friendly communities [9]. Although well intentioned, uptake of the guidelines is challenging given limited dementia care and management in LMICs, coupled with paucity of information about utilization of the toolkit [9].

In 2003, the Uganda government embarked on a countrywide enrollment of lay people (community volunteers) commonly referred to as village health teams (VHTs) to link communities and the health system [10, 11]. The enrolled VHTs were oriented to specific tasks and then attached to health facilities within their communities to support health programs [12]. Their roles included conducting home visits, health mobilization and education, management of common illnesses, follow-up, and community information management [11]. The enrolled VHTs, who in this study we refer to as LHWs, are people in communities who received basic training to support the healthcare system in Uganda by linking communities to formal healthcare, and bridging the gap of insufficient human resources for health in the public sector [2]. Once enrolled into the voluntary service, the LHWs rarely receive refresher training and yet they are a key component in supporting health service delivery nationally.

Uganda's health system is comprised of decentralized healthcare services overseen by district health teams under the leadership of the district health officer who works on behalf of the Ministry of Health. The decentralized district is the local level of decision-making for health services delivery, including the planning and implementation of human resources for health policies, budgeting for medicines, supplies, sundries, infrastructure, and amenities such as electricity and water [13]. Each district has sub-districts, which are lower levels of policy-making and monitoring of health services at the sub-county levels [14, 15]. Each district typically has a general hospital, referral level Health Centers (HC) IV at the health sub-districts, HCs III (with maternal health services and ambulatory care), HCs II (dispensaries), and HCI (community level with village health teams [LHWs]) to provide day-to-day referral of patients from the community [10, 11, 16].

Despite the decentralized healthcare system and the presence of village health teams to link the communities to health facilities, the health of older people has largely been neglected, particularly in rural areas where the majority of the population resides [17]. The prevalence of dementia in adults aged 60 years and over in rural southwestern Uganda is strikingly 20% and needs redress [18, 19]. Building the capacity of LHWs in community-based care and management of people with dementia may help to improve early identification, management, and care of dementia [9]. In this paper, we present results from the pilot intervention of a 5-day training of LHWs in community-based care and management of people with dementia as provided for by the WHO toolkit and feedback after implementing the knowledge and skills gained.

## 2. Methods

### 2.1. Study Design

This was a single district “pre” and “post” pilot intervention study in rural southwestern Uganda to evaluate a 5-day training intervention for LHWs in community-based care and management of people with dementia, and implementation of the knowledge and skills gained. Prior to the intervention, “pre,” we conducted a training needs assessment with the LHWs based on the WHO dementia toolkit to identify their gaps in community-based care and management of people with dementia. We included two additional questions in the interview guide “post” intervention to assess the participants' perceived knowledge and skills gained as well as the experience with the implementation process (Additional file 1). Analysis of the interviews was conducted, and the training needs identified to inform development of an educational intervention.

#### 2.1.1. Educational Intervention and Evaluation

This was a 5-day training intervention of the LHWs that focused on four core competency domains and skills (i.e., understanding dementia; community-based management and care for people with dementia; community engagement; and monitoring and evaluation) as provided for in the WHO toolkit (Additional file 2). The 5-day training was followed by an 8-week (April 29-June 21, 2019) implementation period of the knowledge and skills gained from the training. The intention of our study was not to modify the routine practice of the LHWs by asking them to focus on dementia care, but to enhance their skills to identify and refer old persons with probable signs of dementia within the communities to which they are attached. At the end of the eight weeks, we conducted follow-up interviews to assess the short-term effects of the training on the knowledge and skills of the LHWs. Only the participants who took part in the needs assessment were included in the training and subsequent post-intervention interviews to compare their perceptions “pre” and “post.” We used the same interview guide “pre” and “post”.

#### 2.1.2. Content of the 5-Day Training

(a) Understanding dementia – Common physical and mental conditions among older people, Common beliefs and misconceptions about dementia, What dementia is, Risk factors for dementia, and Other conditions that could be mistaken for dementia; (b) Community-based management and care for people with dementia – How to communicate with people with dementia and their families, How to screen and identify someone who is at risk of dementia, When to refer the person for professional assistance, How to prepare a referral note, How and when to follow up after a referral, How to manage and care for people with dementia, How to support caregivers, and How to care for oneself (the community worker); (c) Community engagement – Memory/Dementia café, Organizing dementia support groups and partners, Public awareness campaigns, and Promotion of safe environments; and (d) Monitoring and evaluation – How to assess the degree of involvement by community leaders, How to tell if awareness of and attitudes about dementia have improved, How to monitor own progress (the community worker), and Checklist for community information and resources

The 5-day training was conducted by a psychiatrist (GZR) from Mbarara Regional Referral Hospital where clients with complications related to dementia are referred for further management. In the Ugandan healthcare system, dementia care and management is provided within mental health services.

#### 2.1.3. Thematic Areas

We adopted the WHO components (i.e., needs assessment, early detection and management, community engagement, and support for people with dementia) as a priori themes for the interview guide. Our analysis involved reporting on results “Pre” and “Post” the 5-day training. We assessed the following:

- Needs assessment. We were assessing the LHWs' understanding of dementia, i.e., their awareness about dementia, and their perceptions about common physical and mental problems of older persons in their communities
- Early detection and management. We wanted to know how the LHWs recognized and managed people with dementia in the community, how they detected signs and symptoms of dementia that required referral to a primary healthcare provider, and how they managed and cared for people with dementia
- Community engagement. We were interested in what the LHWs did in relation to the well-being of the communities that they were attached to, for example, if they visited homes, schools, workplaces, and gathering places, what they did to understand and learn about people's concerns, worries and health conditions
- Support for people with dementia. We were looking for how the LHWs cared for families and people with dementia including engagement, self-care, promoting home safety, and accessing available resources

### 2.2. Study Setting

The study was conducted in Mbarara district approximately 270 km by road, southwest of the capital city, Kampala. Mbarara is the administrative capital of southwestern Uganda with a population of 472,625 of which 242,547 (51.3%) are female. Approximately 19,711 (4.3%) of the population in Mbarara are aged 60 years and over, with 10,787 (54.7%) having a disability [20, 21].

### 2.3. Study Participants and Recruitment

We included one LHW per village from thirty villages in three sub-counties in Mbarara district with the highest number of the elderly population (60 years and above) [22]. We randomly selected ten villages from each sub-county, from where one LHW was identified with the help of local council leaders (gatekeepers to the community) based on their availability to take part in the study, giving an estimated number of 30 participants. All the LHWs provided informed consent to participate in the study.

### 2.4. Data Collection and Analysis

A semi-structured in-depth interview guide was used to collect data from the LHWs in the three sub-counties (10 villages each). The interviews were conducted in the last week of April 2019 using the local language (Runyakore) and backed by field notes.

Data was collected “pre” to identify training needs, and “post” to evaluate implementation of the intervention. All interviews were conducted in person at the locations of the LHWs. Each interview “pre” and “post” lasted approximately 30-45 minutes, was audio recorded, transcribed verbatim by the research assistant, and translated into English. The transcripts were checked by CKK against the audio recordings and validated by EKW for correctness of information and securely stored on a password-protected computer; they can only be accessed by CKK, and EKW.

Coding was done manually, grouping similar quotes together under broad themes while eliminating duplicates, and ensuring inclusion of data from different participants. CO provided a critique of the analysis and interrogated the coding to ensure defendable coding of the data.

The initial coding was done by CKK after discussion with EKW (implementation scientist and qualitative expert), CO and JEH (senior researchers), and GZR a psychiatrist. To address reflexivity, CKK conducted the initial interview together with a trained research assistant to ensure consistency in the conduct of the interviews. The rest of the interviews were conducted by the trained research assistant. CKK conducted the initial analysis of the data and wrote the first draft of the manuscript together with EKW, before sharing with the rest of the authors for their review and input.

## 3. Results

We sequentially present results from the interviews conducted “pre” and “post” the educational intervention.

### 3.1. Participant Characteristics

Thirty participants (20 females and 10 males) from thirty villages in three sub-counties in Mbarara district took part in both “pre” and “post” interviews and the educational intervention. They were aged between 18 and 70 years; 5 participants had completed tertiary education, 16 secondary school, and 9 primary school level.

### 3.2. Theme 1: Needs Assessment

“Pre” training, the LHWs did not think that forgetfulness could be related to a disease although they noted that many elderly people in their communities were forgetting a lot and getting lost from their homes. During the training, the LHWs understood that forgetfulness is a symptom of dementia. “Post” training (implementation phase), they started sensitizing their communities about dementia especially those with elderly persons. Below are examples of responses “pre” the educational intervention, and “post” implementing the knowledge learnt.

“Pre”

*Aah, there are so many things attached… I do not know whether it is a disease or what! But it is a very big problem affecting many people and I do not know how it comes up and I see most elderly people in my village with such a condition, but I do not know how to help them.* LHW 8, Rugando sub-county

“Post”

*Initially we did not know about dementia, but after the training, we went to the homes in our communities to sensitize them that when someone reaches a certain age, they develop a condition of forgetfulness,…I encouraged them to always take them to hospital once they realize the person has developed the condition.* LHW 5, Rugando sub-county

When asked about common physical and mental problems of the elderly persons in their communities, some LHWs attributed getting lost by the elderly persons to poor eyesight while others attributed it to the need to catch up with their long-lost contemporaries. This called for helping them trace their way back to their homes by well-wishers or relatives. After the training and implementation of the knowledge learnt, the LHWs understood that the elderly persons can have other diseases that could potentially contribute to forgetfulness. They also learnt that the elderly can become talkative, although they perceived it as mental illness.

“Pre”

*Someone can just decide to move but with no specific direction looking for their long-lost colleagues…such people are helped by others to find their way back home. When a person gets lost and fails to trace their way back home, it also shows aging because the elderly can somehow get blind.* LHW 5, Bugamba sub-county

“Post”

*They tend to have hypertension, diabetes, urine retention problems...you also see a person becoming mentally retarded and they keep talking to themselves, it might be old age. They can say something now, but when you go back the second time, they will not remember what they told you and they tell something different.* LHW 4, Bugamba sub-county

### 3.3. Theme 2: Early Detection and Management

When asked how they would identify people with dementia or communicate to them before the training, the LHWs noted that in their practice, they were trained to be empathetic, so it was easy for most of them to reach out to the homes in the communities they served and get health-related information about all the family members. Most of the LHWs associated dementia with dirty or unhygienic environments and the fact that caretakers were resigned to helping the elderly who soiled themselves like young children. They however added that they supported such homes with cleaning up the affected elderly persons and then engage in health-related issues. After the training, the LHWs reported improvement in their communication with the family members of the affected elderly persons. They reported that they were able to identify the homes with elderly persons having dementia and that they would work with the family members to ensure that if the elderly person felt unwell, they could easily be referred for healthcare.

“Pre”

*In our general practice and training as LHWs, we were told to always help the needy in every possible way. As a VHT [lay health worker] member, when I go to visit homes and I find the elderly people in a dirty environment with soiled and unwashed clothes, I first ensure that they are cleaned up to improve their hygiene before I ask about their health. You find that many times those people [the elderly] are sick but their relatives have given up on them because they are behaving like children.* LHW 2, Ndeija sub-county

“Post”

*…like the one I visited, because I knew she had dementia, I reminded her of what we had talked about during my previous visit to check if she could remember anything. After that I told her about going to the hospital if she felt unwell, I also talked to a family member that if the forgetfulness of the old woman persists, she should come for a referral letter to the health facility. I try to counsel the families I visit generally.* LHW 1 Ndeija sub-county

In relation to how the LHWs determined when to recommend referral for healthcare, most of the participants related experiences around them to do with forgetfulness in daily living.

“Pre”

*… these people forget a lot for example, I have an elder brother who placed a smoking pipe in the mouth not wanting to put it down to avoid dirt from falling in, so he went to the plantation, and he came back home looking for the smoking pipe not knowing it was in his mouth.* LHW 8, Rugando sub-county

“Post”

*Now, if a person has always been going to meetings and he/she comes and fails to submit his/her views as before, you begin to ask yourself what went wrong since a person used to be active during meetings. …you may find that this person is developing dementia, so you refer for medical attention*. LHW 4, Ndeija sub-county

### 3.4. Theme 3: Community Engagement

Our interaction with the participants at the two time points “pre” and “post” revealed that the LHWs perform general roles ranging from sanitation, health education in several areas including the need for good nutrition, taking children for immunization, pregnant mothers attending antenatal care, among others. The LHWs go to homes as well as places of worship and schools. Before the training, participants did not describe inclusion of dementia care in these activities. After the training, some participants were able to introduce aspects about dementia and community management in the health education sessions as exemplified below:

“Pre”

*My role to the households is health educating them about their health and sanitation. I tell them how they are supposed to look after themselves for example, I tell pregnant women about antenatal care and immunization of their children. Another thing, I teach them how to sustain themselves because there is need to work for your old age when you are still young. It is my role to tell them all these… I also go to schools in the community and teach them about sanitation, and how to relate between teachers, parents, and pupils, because if there is no good relationship, there are challenges.* LHW 10, Bugamba sub-county

“Post”

*When I am doing sensitization in the trading centers, homes and even schools, I try to bring in examples from all diseases whether its cancer, HIV, or even dementia. I encourage people to treat patients with dementia like normal people because we all need each other to live well in the community. When it comes to communicable diseases, I tell the people to be careful with the sharp things that they use, let us say a razor blade, they should not leave it in the open or share with anyone, but dispose it like in a pit latrine when they do not need it*. LHW 3, Rugando sub-county

We found no specific support for people with dementia in the community. This perception was noted both “pre” training the LHWs on community-based management of dementia and “post” the training. The support groups that most of the participants referred to were community burial groups with nothing to do with dementia care. However, there was a difference in the perceptions after the training where the participants indicated that they encouraged family members to take their people with dementia to the health facilities because speaking with the healthcare providers was support in itself. They however acknowledged that support for dementia was limited because of inadequate knowledge about the condition.

“Pre”

*Do you think we have that kind of community support? I do not think so, that would be coming from social support groups like the ‘twezikye' [let us bury ourselves] …but still these do not help sick people, instead they help during burial arrangements.* LHW 10, Ndeija sub-county

“Post”

*There is no direct support for the elderly people since the community does not know that forgetfulness is a disease. We started visiting and talking to families struggling with elderly people after the training to give them hope and help refer the elderly to the health centers to talk to the doctor. Talking to a doctor or health worker just makes them feel better.* LHW 3, Bugamba sub-county

### 3.5. Theme 4: Support for People with Dementia and Their Family Members

We found that the support that the LHWs provided especially to households with elderly people was no different than what was required of them for dementia care. The participants reported paying more attention to families with elderly people either living alone or those with young grandchildren so that they are with no assistance at any one time in case of any health emergency. One of the functions of the LHWs was to ensure proper sanitation for every household regardless of whether there were elderly persons living there or not. The LHWs reported that, for the families where the elderly people were living with older grandchildren but lacked proper basic care, they provided instructions on self-care including constructing pit latrines for proper human waste disposal, home safety, and proper nutritional feeding. They indicated working with local council leaders for support in implementation of their recommendations, ensuring that there were minimal challenges. The participants indicated that they had not received specific training in dementia care prior. Below are examples of responses “pre” and “post” the training:

“Pre”

*…sometimes these elderly people stay with grandchildren, let us say when there are mosquito nets to distribute, we give these families the first chance to benefit. When these families of elderly people do not have toilets, we take the responsibility to tell the older children to construct them for better hygiene. We know the homes of the elderly that do not have older children staying with them, we always check on them to make sure that they do not get sick and if they do not have assistance…it is our responsibility of visiting them either every morning or evening. We tell the families about self-care and the need to eat a balanced diet. Concerning security, we ensure that there is always someone to watch over them.* LHW 1, Ndeija sub-county

“Post”

*I encourage the caretakers to always accompany the elderly people with dementia especially when they want to move out because they are vulnerable and should not be left alone in case they go to wrong places and get lost. Concerning personal hygiene, we ensure that families have clean boiled water stored in clean containers. We also mobilize ourselves as LHWs and inform the chairman about inspecting the villages where we demolish poorly built latrines and encourage them to have well maintained pit latrines. We work hand in hand with the local council leaders. We provide basic information based on experience, [prior to this study] we had never had any training concerning dementia and how we should handle it in the community until now.* LHW 5, Rugando sub-county

### 3.6. Theme 5: Evaluation of the Eight Weeks Implementation Phase

With the preliminary training, we were interested in the participants' perceived knowledge and skills gained in community-based care and management for people with dementia as well as experiences during the eight weeks implementation phase.

#### 3.6.1. Perceived Knowledge and Skills Gained

When asked about the knowledge and skills gained, some participants noted that their understanding and attitude towards dementia had changed, and that new knowledge had been added to them. Prior to the training, most of them did not know that dementia was an illness; however, after the training, their knowledge improved, and they reported ability to explain to the communities that “forgetfulness” was a disease. Importantly, they acknowledged the need for more training in dementia care and management to confidently support the communities they served.

*We never knew that forgetfulness in old people was a disease, so we did not pay attention to it when we were sensitizing the communities to take patients for healthcare. When they called us for the training, we learnt new things and understood that it was a medical condition that needed attention. But we have not acquired sufficient skills in dementia care that will enable us to work independently and help our people.* LHW 4, Bugamba sub-county

Like anybody else in the communities, the participants associated forgetfulness with witchcraft. After the training, participants noted that their perceptions changed and that they had learnt how to handle people with dementia and their families.

*I learnt how to approach these people's homes for example we were taught to ask few questions that will not overwhelm them. Previously, before the dementia training, people used to relate dementia to witchcraft but today when they see me, they say “oh have you come to tell us about our disease of forgetfulness?”* LHW 6, Ndeija sub-county

The participants reported that although they were making home visits to different communities, they viewed the elderly persons with dementia as a burden and they did not know how to support the families. However, after the training, they felt they could now sensitize the communities on what dementia is and what it is not, as exemplified below:

…*now the situation is better than before, we used to look at these people as a burden but after training, it is now better. We now sensitize the caregivers and they have come to accept that dementia is a disease. We call village meetings and talk to people that dementia is a disease that comes with old age… in the past people thought it was insanity, family stress, or picky behavior of the person.* LHW 5, Rugando sub-county

#### 3.6.2. Experiences during Implementation

The participants found it challenging to differentiate the signs and symptoms of early dementia, such as forgetfulness, self-neglect, and wandering about, as a disease from superstitious beliefs, such as witchcraft. In the very first visit to the community after the training, most of the participants acknowledged experiencing a difficult time articulating what dementia was. They reported difficulty in explaining the cause of behavioral changes due to varied perceptions and beliefs in the community. They noted that some people believed them, while others remained in doubt.

*It was difficult…you know how the village people can be and their beliefs, they would say ‘aah how can forgetfulness be a disease? It is witchcraft!' … it took me a lot of time to explain what dementia was, ehhh! Some people understood but others did not believe that forgetfulness was a disease.* LHW 10, Rugando sub-county

It was therefore complicated for some participants to convince the families with elderly people suffering from forgetfulness to take the patients to a health facility for a dementia diagnosis. They noted that most people in their communities tended to seek medical attention when they had a physical ailment and were bedridden.

*For a ‘normal' person [with no physical ailment] who is not yet been bedridden, it was so difficult for them to go to the hospital. The elderly person would even think that ‘maybe they want to kill me from there'…it was difficult for them to go to the hospital after referring them for a dementia diagnosis.* LHW 5, Ndeija sub-county

For the family members who complied to the referral and took their elderly persons with challenges of forgetfulness to the nearest health facility, the participants reported that the family members returned with no assistance because the health facilities where they went did not offer dementia-related services, thus requiring onward referral.

*I have tried to refer them to the nearest health facility but unfortunately, they bounce and come back because there are no services; when they reach the facility and explain the problem, the health workers tell them that ‘for us we do not treat such cases here' so they come back without any help*. LHW 4, Rugando sub-county

On the positive side, the participants reported improvement in how they handled the families and/or the people with dementia and the fact that their support was appreciated. They however expressed the need for more training in dementia care and management to better support the communities.

*If we could be empowered with more skills [in dementia care], people can live to testify that we are important in their lives by what we have done with the little knowledge gained. …through our coordinator, more trainings can be organized to keep us updated and improve the way we deliver our care services.* LHW 6, Ndeija sub-county

Participants also reported a notable change in the attitude of the family members towards the people with dementia in the communities. They noted that initially, the family members thought that the elderly persons intentionally behaved in annoying ways, but after the sensitization, which included educating the communities about aging and the associated complications, the family members' attitude improved and there was better care.

*I have noticed a change in the care of people with dementia because the attitude of caregivers and families is better. Previously they thought that these elderly people had strange mannerisms and intentionally did the things that annoy. This has reduced, people have started recognizing that the behavior started with old age*. LHW 4, Bugamba sub-county

## 4. Discussion

This was a “pre” and “post” qualitative pilot intervention study examining the ability of LHWs to support community-based care for people with dementia in rural southwestern Uganda where the prevalence of dementia in adults aged 60 years and over is 20% [18, 19]. Many more people are expected to reach old age in the next decade; it is therefore important to envision how the Uganda's population can grow older with dignity [23]. Given that Uganda uses the approach of working with LHWs to support healthcare delivery [10, 11] enhancing the capacity of LHWs is necessary although lacking. LHWs have in-depth knowledge of a community and understand cultural practices better than formal healthcare providers, which may make them better placed to support community-based care and management for people with dementia [24].

Although the LHWs may not have much formal education, they can be trained to provide selected health services to offer support to related healthcare delivery [25, 26]. In this study, we identified knowledge gaps in community-based care and management as reported by the LHWs, provided a 5-day training, allowed the LHWs eight weeks to implement the knowledge and skills gained, and then assessed their experiences with providing support in dementia care and management.

The LHWs had significant knowledge gaps in what dementia was and the basic signs they could use as indicators to link the communities to healthcare. During the training, the LHWs were surprised to learn that forgetfulness is a sign of dementia, but they were able to relate with it very well and showed readiness to sensitize their communities. At the point of implementation, it was a challenge for the LHWs to convince some members in the communities who had superstitious beliefs that forgetfulness was witchcraft. However, other community members understood, and they were able to change attitudes. Further training for the LHWs will likely improve their ability to educate and convince even those who are reluctant to believe in dementia.

Our study suggests that mental healthcare (where dementia is categorized in the Uganda health system) may be supported through trained and supervised LHWs [3, 27]. This potential is key because one major barrier to appropriate dementia diagnosis and treatment in low-resource countries is the lack of specialist. There is great potential and expectation that primary care physicians and non-specialists such as LHWs can play a much bigger role in identification and management of people with dementia [9]. In the Ugandan healthcare system, evidence shows that LHWs (village health teams) are instrumental in improving healthcare delivery at the community level including immunization and malaria campaigns [11]. Once their capacity is built, it does seem possible that LHWs may play a crucial role in community-based identification and care for people with dementia [28, 29].

Previous studies have shown inadequate knowledge about early signs and symptoms of dementia among health workers as a barrier to achieving early detection and a timely diagnosis of dementia [30]. From our interaction with the LHWs in this study, early signs of dementia were characterized as simply elderly living in dirty or unhygienic environments. The LHWs were addressing the hygiene situation because it was part of their training and responsibilities. These interventions were never associated to be supporting elderly with dementia, neither was aging or forgetfulness considered a disease. There is clearly a need and opportunity for comprehensive training of the LHWs on the signs and symptoms of dementia to facilitate early detection which can lead to earlier access to treatment, care, and prevention support. This in turn can help better manage the disease and enhance the quality of life for people with dementia and their carers [31, 32]. It also gives the individuals, their families, and their communities time to set up support networks to help in the ongoing care of the patient [9]. Managing people with dementia will require continuous training and refresher courses among health workers [33, 34].

According to the WHO guide for community-based management and care for people with dementia, a community worker is concerned with the well-being of the whole community. It is therefore expected of them to personally visit homes, schools, workplaces, and gathering places to understand and learn about people's concerns, worries, and health conditions, sharing with them good habits and practices that promote physical and mental health [9]. While the LHWs in our study performed most of these functions, they did not pay specific attention to dementia. This gap can be largely attributed to no specific training in community-based care and management for people with dementia. The need for training on dementia extends to community members, healthcare providers, and families so that holistic support and care can be provided [34, 35]. There are currently no formal support structures in the rural areas for dementia care, not even at the lower health facilities where the LHWs refer patients for further management. It was unfortunate to hear from participants in this study that the people with dementia who were referred to health centers for support were not attended to because the primary healthcare providers at the lower facilities were not trained to provide dementia care. This finding is consistent with a study in the UK that reported the need to train general practitioners in the diagnosis and management of dementia [36].

The support LHWs provides to communities in their day-to-day engagements is not very different than what would be expected of them as guided by the WHO toolkit for community workers in LMICs including general care of older people (eating and drinking), getting around, grooming (dressing, bathing), and sometimes toileting and continence care. The difference is that for dementia care, the LHWs need extra training or skills in engaging the family, encouraging independence and self-care, promoting home safety, and managing changes in mood, behavior, and personality of people with dementia. With dementia, there is a decline in memory and functional abilities including changes in mood, agitation, aggression, repetitive actions, wandering or getting lost, and poor judgement. The family members will need to know how to manage locally when such instances happen. Holistic training for workers is needed to provide improved and sustainable dementia care and management at community level [37].

Our finding that early signs of dementia are confused with superstitious beliefs, such as witchcraft, is aligned with other dementia studies in southwestern Uganda, Tanzania, and South Africa [38–40]. Notable, however, during the interviews was an obvious level of discomfort by the participants when narrating their experiences during the implementation phase. The LHWs in this study found it challenging to differentiate the signs and symptoms of early dementia and superstitious beliefs. It was not yet natural for them to believe that behavior changes in old age could be related to dementia and not necessarily witchcraft. The knowledge about dementia was new for the participants. They will need more training and time to better equip them with deeper knowledge and skills to be effective change agents in the support of community-based care of people living with dementia.

## 5. Limitations

Our study was designed to assess the short-term impact of the training on the LHWs and to understand their experiences with community-based management of people with dementia. This study was not designed to assess the objective impact of the LHWs on the quality of life of community members or specific aspects of dementia care as no support supervision was provided during the 8-week period when the LHWs implemented the knowledge and skills gained from the training. Supervision would be important for future adoption of training programs such as was carried out in this study.

Additionally, the fact that study participants were aware of age-related problems such as dementia among the elderly, even though the term was new to most of them, and the fact that they were involved in the study about dementia we cannot therefore rule out the methodological effect of this knowledge (Hawthorne effect) [41] on the study outcomes.

This was a single district pilot intervention study with one LHW per village from thirty villages in three sub-counties and therefore may not be generalizable to other districts. The results, however, will be useful for a larger study to confirm the effectiveness of utilizing LHWs to support community-based management of dementia.

## 6. Conclusion

With the 5-day training in this pilot intervention study, the participants indicated a change in knowledge and attitude towards dementia validating findings in other studies that with adequate training it is possible to obtain effective support from LHWs [3, 27]. Our findings suggest that enhancing the capacity of LHWs in rural settings to support community-based care and management for people with dementia may be a viable option especially given the scarcity of professional healthcare providers. Our study demonstrated that a simple training intervention can result in observable changes in the attitudes and actions of LHWs and empower them to play a crucial role in educating family and community members. Further research is needed to determine the best approach to scale, evaluate, and sustain this approach to community-based care for dementia in LMIC settings.

## Data Availability

Data is not publicly available because our work is still ongoing but will be available in future when the main study is completed.

## Abbreviations

LHWs: Lay health workers
LMICs: Low- and middle-income countries
MADRI: Mbarara Alzheimer's disease research initiative
MUST-REC: Mbarara University Research Ethics Committee
UNCST: National Council for Science and Technology
WHO: World Health Organization
HC IIs: Health Centre IIs.

## Supplementary Materials

Supplementary Materials Additional file 1: In-depth interview guide. Additional file 2: Components of the educational intervention.
