# Supplementary material for: Lay Health Workers in Community-Based Care and Management of Dementia: A Qualitative ‘Pre' and ‘Post' Intervention Study in Southwestern Uganda
Source: Biomed Res Int. 2022 Mar 23;2022:9443229. doi: 10.1155/2022/9443229 (PMC8967551; doi:10.1155/2022/9443229)
Supplement: Supplementary Materials — Additional file 1: In-depth interview guide. Additional file 2: Components of the educational intervention. [file 9443229.f1.docx]

# Additional file 1: IN-DEPTH INTERVIEW GUIDE

**DEMOGRAPHICS**

1. Gender
2. Age
3. Level of Education
4. Sub-county
5. Parish
6. Village/cells
7. Number of households

**NEEDS ASSESSMENT**

1. What would you say about someone who forgets a lot?

*Probe for*

*a) Losing track of time*

*b) Becoming lost in familiar places*

*c) What they would do to help*

1. What are the common physical and mental problems of older people in your community?

*Probe for*

*a) Common physical problems for older men/women*

*b) Common mental problems for older men/women*

**EARLY DETECTION AND MANAGEMENT**

1. How do you identify someone with dementia (disease of forgetfulness) and how would you communicate to them?

*Probe for*

*a) Talkativeness*

*b) Body language (smiles, hugs, touching hands)*

*c) Talking to family member(s) or caregiver(s) (number of times)*

*d) Listening to stories from the person with dementia and their family members*

1. How do you know that some needs a referral to a primary healthcare facility for special attention?

*Probe for the signs they use*

**COMMUNITY ENGAGEMENT**

1. As a community worker, you are concerned with the well-being of the whole community. Please tell me about your roles to the households you are attached to?

*Probe for*

*a) Visits in homes, schools, workplaces*

*b) What they do during the visits in terms of people’s concerns, worries and health conditions*

**SUPPORT FOR PEOPLE WITH DEMENTIA/FAMILY MEMBERS**

1. How do you support the care for people with dementia?

*Probe for*

*a) Engaging the family*

*b) Encouraging self-care (eating, bathing, personal hygiene)*

*c) Promoting home safety*

*d) Accessing available resources (like church, support networks)*

**ADDITIONAL QUESTIONS POST INTERVENTION**

1. *What skills did you gain from the training?*
2. *Tell me about your experience during the 8 weeks of implementing what you were taught*

**Additional file 2: Components of the educational intervention**

| **Core Competency Domains and Skills** | | |
| --- | --- | --- |
| **Days of training** | **Domain** | **Core competencies** |
| Day 1 | Understanding Dementia | - Common physical and mental conditions among older people - Common beliefs and misconceptions about dementia - What dementia is - Risk factors for dementia - Other conditions that could be mistaken for dementia |
| Day 2 | Community based management and care for people with dementia | - How to communicate with people with dementia and their families - How to screen and identify someone who is at risk of dementia - When to refer the person for professional assistance - How to prepare a referral note |
| Day 3 |  | - How and when to follow up after a referral - How to manage and care for people with dementia - How to support caregivers - How to care for oneself (the community worker) |
| Day 4 | Community Engagement | - Memory/Dementia café - Organizing dementia support groups and partners - Public awareness campaigns - Promotion of safe environments |
| Day 5 | Monitoring and Evaluation | - How to assess the degree of involvement by community leaders - How to tell if awareness of and attitudes about dementia have improved - How to monitor own progress (the community worker) - Checklist for community information and resources |
